# Editorial: Women in plant multi-omics: 2023

**DOI:** 10.3389/fpls.2024.1487736

**Published:** 2024-09-25

**Authors:** Min Tu, Yanmei Chen, Weizhen Liu, Qingyi Yu, Yin Li, Wenqin Wang

**Affiliations:** ^1^ Hubei Technical Engineering Research Center for Chemical Utilization and Engineering Development of Agricultural and Byproduct Resources, School of Chemical and Environmental Engineering, Wuhan Polytechnic University, Wuhan, China; ^2^ State Key Laboratory of Plant Environmental Resilience, College of Biological Sciences, China Agricultural University, Beijing, China; ^3^ School of Computer Science and Artificial Intelligence, Wuhan University of Technology, Wuhan, Hubei, China; ^4^ Daniel K. Inouye U.S. Pacific Basin Agricultural Research Center, Agricultural Research Service, United States Department of Agriculture, Hilo, HI, United States; ^5^ The Genetic Engineering International Cooperation Base of Chinese Ministry of Science and Technology, Key Laboratory of Molecular Biophysics of Chinese Ministry of Education, College of Life Science and Technology, Huazhong University of Science and Technology, Wuhan, China; ^6^ College of Life Science, Shanghai Normal University, Shanghai, China

**Keywords:** women in science, STEM, multi-omics, genomics, transcriptomics, proteomics, metabolomics

Welcome to this Research Topic of Women in Plant Multi-omics 2023. Frontiers in Plant Science is proud to offer this platform to highlight the work of women scientists across all fields of plant multi-omics and to hopefully inspire the next generation of female scientists.

Equal opportunities must be offered to females who pursue careers in science, technology, engineering and mathematics (STEM) disciplines, and long-standing biases and gender stereotypes in science-related fields should be defeated. According to the UNESCO Science Report 2021, women remain a minority in the field of science, with female researchers making up only 33% of the global research population. Persistent biases and gender stereotypes discourage females from entering science-related fields, particularly STEM research. UNESCO has emphasized that science and gender equality are essential for sustainable development (Baird, 2018; Fisher et al., 2020). In some countries, a series of actions and policies have been implemented to promote women in science. In 2021, the Chinese Ministry of Science and Technology, together with 12 Chinese government departments, announced measures to support female talent in playing a greater role in the innovation of science and technology. Moreover, the Women’s Association of China for Science and Technology has collaborated with the United Nation Women for years, holding the “Symposium on Empowering Young Female Scientists” to help create a positive atmosphere with equal gender opportunities.

In terms of facilitating change, Frontiers in Plant Science believes that gender equality can be fostered through this special Research Topic. Undoubtedly, female scientists have made significant achievements in the field of plant multi-omics in all its diversity. As such, the present Research Topic has accepted for publication five representative contributions, including three research articles, one opinion, and one review. These works highlight the diversity of research performed across the entire breadth of plant multi-omics, as well as the diversity of countries ranging from China to Italy to the United States of America.

Chen et al. identify the gene families encoding the switch defective/sucrose non-fermentable (SWI/SNF) multi-subunit complex in sorghum. The authors portrayed the expansion of sorghum *SWI/SNF* families and uncovered a positive relationship between nucleosome phasing and gene expression during sorghum development and responses to the osmotic stress by integrating comparative genomic, transcriptomic, and epigenomic approaches.

Yang et al. focused on cepharanthine (CEP), an important bisbenzylisoquinoline alkaloid (bisBIA) compound extracted from the traditional Chinese medicine *Stephania japonnica*, which is known for its anti-coronavirus properties. Using the *Stephania japonnica* genome, the authors characterized 59 genes encoding the ethylene response factor family. A *SjERF* gene cluster was found on chromosome 2, containing three *SjERF*s, which were associated with CEP metabolic genes. RNA-seq expression profiling and coexpression analysis helped to identify 13 *SjERF*s that were highly expressed in the root and correlated with the contents of alkaloids and CEP biosynthetic genes. This work provides valuable information for improving the economic value of *Stephania japonnica*.

Qiao et al. identified genes regulating color formation in Chinese red chestnut and explored its nutritional value. Metabolic profiling determined six major anthocyanin compounds in the chestnut fruits. Combining metabolic and transcriptome data allowed for the identification of several pigment biosynthetic genes (e.g., CHS, CHI, F3H, CYP75A, and CYP75B1) with expression patterns associated with anthocyanin dynamics. Coexpression analysis further revealed four WRKY transcription factors potentially involved in the color formation of red chestnut. These findings provide a valuable gene resource for chestnut improvement.

Roman-Reyna et al. reported their opinions on the importance and application of metagenomics in crop research. The authors indicated that metagenomics could play a critical role in understanding plant pathogens and their interactions with host crops. A best practice workflow for metagenomic experiments was also proposed and discussed, and state-of-the-art resources of metagenomics were reviewed and summarized for the research community. Moreover, future directions were discussed, focusing on the application of metagenomics—on its own or in combination with other multi-omics—to boost crop research and sustainable agriculture.

Janni et al. focused on global warming as one of the most impactful abiotic stresses that may severely affect future international food security. To mitigate the threat of impact of global warming, the authors discussed the relationship between global warming and its impact on the sustainability of natural and agricultural ecosystems. They reviewed recent advances in the mechanisms of crop responses and resilience to heat stress enabled by omic technologies, including genomics, proteomics, metabolomics, phenomics, and ionomics. Additionally, the authors emphasized the use of resource-saving technologies, such as precision agriculture and new fertilization technologies to improve the tolerance and adaptability of crops to global warming. Overall, this comprehensive review lays the basis for new research paradigms that not only consider crop yield, but also multiple agricultural aspects including sustainability, agroecosystem management, and commercialization.

Finally, the editors and topic coordinators would like to express our gratitude to the contributors and reviewers. Without their contribution and efforts, this Research Topic would not have been possible. Plant multi-omics is a multifaceted discipline that requires a diverse group of experts and minds to solve the next generation of questions. Therefore, we believe that ensuring gender equality is of great importance to achieving the involvement of a multifaceted group of scientists in plant multi-omics research.
